# The Finger of the State

**Published:** 1924-06

**Authors:** 


THE
### HOSPITAL and HEALTH REVIEW

### THE FINGER OF THE STATE.

"The time has come for a survey of the whole field of hospital activity." So wrote the Prime Minister in his letter to the promoters of the Conference on "The Hospital Problem," which was recently organised by the Labour Party. That Conference, which enjoyed the participation of hospital, medical, Labour, and other representatives, was highly interesting and of real value as bringing out clearly two widely differing ideals of hospital polity. In the sentence we have quoted Mr. MacDonald rather understated the position, since the survey of which he speaks has been in operation at the hands of hospital leaders ever since the beginnings of the searchings of heart produced by the after-war problems by which the voluntary system was faced. On the other hand, his description of our hospital system as "chaos" was distinctly an over-statement. Nowhere is there such acute consciousness of the shortcomings of that system as in the minds of the men responsible for its working. They are the first to agree that "the needs of the time demand the application of the scientific spirit, the co-ordination of effort, and the elimination of waste." The Prime Minister paid handsome testimony to "the labours of those great public servants" who have made our hospitals what they are, while hinting, not obscurely, that they must themselves be dissatisfied with the situation. Yet it is impossible not to feel that there is a distinct *parti pris* in the Prime Minister's mind.

The Conference naturally turned in great measure upon the inadequate accommodation of the voluntary hospitals. The Labour view is that, if proper provision is to be made 160,000 beds are needed, whereas the number actually available is less than one-third of that figure. On the other hand, Mr. Harman pointed out that, including, we presume, the Poor Law Infirmaries, there are actually 362,000 beds in the United Kingdom, a total at which it is impossible to cavil. As regards the voluntary hospitals, taken by themselves, the deficiency is notorious, as Lord Knutsford admitted. The simple truth is that the Infirmaries belonging to the Guardians are not pulling their weight, and that if they were thrown into the general hospital pool there would be little, if anything, to complain of in the matter of accommodation for patients, and it was of this that the Conference was obviously thinking when it passed a resolution that, while developments should be directed to preserving what is best in the present voluntary system, "some form of public assistance is essential if a complete and adequate hospital system is to be maintained." It would be at once absurd and extremely wasteful to continue to maintain two systems side by side, one purely voluntary, the other charged upon the rates. But if the Poor Law Infirmaries are to be thrown into the general hospital system it is essential, as we have often pointed out, and as is now universally recognised that they should, in the words of the Conference resolution, be "removed from all taint of the Poor Law." No man, woman, or child who requires hospital treatment ought any longer to be branded as a pauper because there is nobody to pay the cost. The argument put forward by Dr. Somerville Hastings, M.P., who outlined the Labour Party's case, that payments made by potential patients "tend to act as a deterrent, and threaten to change hospitals into nursing homes for the middle classes," was not only absurd in itself but indicated lack of knowledge of the facts. It is not true that money for the hospitals is anywhere "deducted" from wages. The word suggests compulsory deduction by the employer, which is very far indeed from the truth.

Lord Knutsford had no difficulty in showing that the point of this argument is exceedingly blunt; at the London Hospital, indeed, 43 per cent. of the patients pay nothing for the sufficient reason that they cannot. That the voluntary hospitals lack beds is the one substantial accusation against them, but to hand them over to State management and control as the only means of adding to their accommodation would be to repeat on a colossal scale the immortal expedient of burning down your house to roast your pig. The new methods of hospital finance need a much more extensive trial before we come to the conclusion that voluntaryism has had its day. That is not to say that modifications of the system may not be necessary, and Lord Knutsford made the interesting suggestion that there should be a sick rate for everybody, assessed according to income. The proposal needs elaboration, and more will perhaps be heard from Lord Knutsford on the subject. It has the value of a middle course, though it is impossible not to entertain apprehensions as to the nature of the State claims that might follow, to say nothing of the probable effect of a compulsory sick rate upon the great Hospital Funds. The only thing that can be predicted with certainty of State management of hospitals is that it would be expensive. It might, and probably would be, less efficient than the existing system which, with all its defects—every one of them remediable—has achieved marvellous things and lighted a beacon wherever the promptings of Christian compassion have obtained entrance. The voluntary hospital is, indeed, mankind's noblest monument, and to pull it down would be not the least of the world's historic tragedies.

